# Characterization of exosomes in peritoneal fluid of endometriosis patients

**DOI:** 10.1016/j.fertnstert.2019.09.032

**Published:** 2020-02

**Authors:** Hannah M. Nazri, Maria Imran, Roman Fischer, Raphael Heilig, Sanjiv Manek, Rebecca A. Dragovic, Benedikt M. Kessler, Krina T. Zondervan, Thomas T. Tapmeier, Christian M. Becker

**Affiliations:** a Endometriosis CaRe Centre, Nuffield Department of Women’s & Reproductive Health, University of Oxford, Oxford, United Kingdom; b Nuffield Department of Medicine, University of Oxford, Oxford, United Kingdom; c Department of Cellular Pathology, Oxford University Hospitals, Oxford, United Kingdom; d Wellcome Centre for Human Genetics, University of Oxford, Oxford, United Kingdom

**Keywords:** Biomarker, endometriosis, exosomes, pathogenesis, peritoneal fluid

## Abstract

**Objective:**

To demonstrate the feasibility of studying exosomes directly from peritoneal fluid, we isolated exosomes from endometriosis patient samples and from controls, and characterized their cargo.

**Design:**

Case-control experimental study.

**Setting:**

Academic clinical center.

**Patient (s):**

Women with and without endometriosis who underwent laparoscopic surgery (n = 28 in total).

**Intervention (s):**

None.

**Main Outcome Measure (s):**

Concentration of exosomes within peritoneal fluid and protein content of the isolated exosomes.

**Result (s):**

Peritoneal fluid samples were pooled according to the cycle phase and disease stage to form six experimental groups, from which the exosomes were isolated. Exosomes were successfully isolated from peritoneal fluid in all the study groups. The concentration varied with cycle phase and disease stage. Proteomic analysis showed specific proteins in the exosomes derived from endometriosis patients that were absent in the controls. Five proteins were found exclusively in the endometriosis groups: PRDX1, H2A type 2-C, ANXA2, ITIH4, and the tubulin α-chain.

**Conclusion (s):**

Exosomes are present in peritoneal fluid. The characterization of endometriosis-specific exosomes opens up new avenues for the diagnosis and investigation of endometriosis.

## Abstract (es)

Caracterizacion de exosomas en el fluido peritoneal de pacientes con endometriosis

**Objectivo:**

Para demostrar la viabilidad de estudiar de exosomas directamente del líquido peritoneal, aislamos exosomas de muestras de pacientes con endometriosis y de controles y caracterizamos su carga.

**Diseño:**

Estudio experimental caso-control.

**Lugar:**

Centro clínico académico.

**Pacientes:**

Mujeres con y sin endometriosis que se sometían a una laparoscopia quirúrgica (n=28 en total).

**Intervención:**

Ninguna.

**Resultados principales:**

Concentración de exosomas dentro del fluido peritoneal y contenido proteico de los exosomas aislados.

**Resultados:**

Las muestras de líquido peritoneal se agruparon de acuerdo con la fase del ciclo y el estado de la enfermedad para formar seis grupos experimentales, de los cuales se aislaron los exosomas. Los exosomas fueron exitosamente aislados del líquido peritoneal en todos los grupos de estudio. La concentración varió según la fase del ciclo y el estado de la enfermedad. El análisis proteómico mostró proteínas específicas en los exosomas derivados de pacientes con endometriosis que estaban ausentes en los controles. Se encontraron cinco proteínas exclusivas del grupo con endometriosis: PRDX1, H2A tipo 2-C, ANXA2, ITIH4 y la cadena a de tubulina.

**Conclusiones:**

Los exosomas están presentes en el fluido peritoneal. La caracterización de exosomas específicos de endometriosis abre nuevas vías para el diagnostico y la investigación de la endometriosis.

**Discuss:** You can discuss this article with its authors and other readers at **https://www.fertstertdialog.com/users/16110-fertility-and-sterility/posts/53994-28445**

Endometriosis affects millions of women of reproductive age worldwide (1), with a minority of women continuing to experience endometriosis even into menopause (2). The symptoms include menstrual and non-menstrual pain that is often aggravated during and after coitus, defecation, and micturition. Additionally, up to half of women with endometriosis experience a degree of infertility as well as mental health issues and fatigue (3). Consequently, endometriosis not only affects women and their partners but society in general, with ever-rising health care and non–health care costs (4). Furthermore, due to the “unhappy endometriosis triad” of insufficient public and professional awareness (5), lack of clinically relevant biomarkers (6), and the nonspecificity of endometriosis-associated symptoms, women wait on average for 6 to 9 years (7) before the definitive diagnosis is made by laparoscopy (8).

Endometriosis presents as ectopic endometrial-like lesions mostly in the pelvis along the peritoneum, at times involving surrounding structures, or as endometriomas, or as fibrotic nodules (1). Pelvic endometriosis is classified into stages I–IV (minimal to severe) based on direct visualization during surgery (9). The current endometriosis treatments are not curative and are targeted toward symptom amelioration and restricted to hormone treatment or surgical excision while also aiming for fertility preservation if required (10). Although retrograde menstruation is thought to be the underlying cause for most intra-abdominal endometriosis (11), more than 90% of women have menstrual blood in their peritoneum during menses (12). The reason why endometriosis develops only in some women remains unknown, and the search for noninvasive tests (13) and blood biomarkers (14) has been unsuccessful so far.

Recently, exosomes have gained considerable interest as novel agents of intercellular communication in health and disease (15): Exosomes are cell-derived lipid-bound nanovesicles secreted by virtually every cell, so they are present in almost all biological fluids (16). They are formed within multivesicular endosomes (MVE) and released by the fusion of the MVE with the plasma membrane of the cell (16). Proteins like Alix, CD9, and syntenin are instrumental in exosome biogenesis and thus serve as markers in exosome enrichment and purification protocols.

Tumor-derived exosomes have been shown to direct metastasis by preparing the premetastatic niche (17), and exosomes have been described in benign diseases such as obesity and diabetes (18), preeclampsia (19), in hematologic (20) and neurodegenerative diseases (21), and in atherosclerosis (22) and coronary artery disease (23).

Exosomes carry surface markers, genetic material, peptides, and proteins from their parent cell, which mirror its gene and protein expression (16). This makes exosomes an attractive candidate as a diagnostic and therapeutic tool. Exosomes have been evaluated as biomarkers of pancreatic cancer (24); in coronary artery disease, disease-specific circulating exosomes have been shown to add prognostic value to an existing scoring system and to increase the predictive value of a risk factor model for major adverse events (23). At the moment, more than 20 clinical trials involving exosomes are recruiting participants (clinicaltrials.gov).

In endometriosis, an in vitro study (25) showed that exosomes isolated from cultured endometrial stromal cells of endometriosis patients carried mir-21, a microRNA implicated in angiogenesis. Other studies have similarly investigated exosomes derived from serum (26) or cultured endometriotic material (27, 28). However, as opposed to the indirect sourcing of exosomes, no direct analysis of the exosomes presumably present within the peritoneal fluid (PF) of women has yet been undertaken. Here, we directly detect exosomes in the PF of women and begin to characterize the PF-derived exosomes with regards to their protein cargo.

## Materials and methods

The samples and clinical data were obtained as part of the larger prospective ENDOX study of endometriosis biology at the Oxford Endometriosis CaRe Centre (29), approved on July 13, 2009, by the Oxford University Hospitals Foundation Trust, with ethics approval by the South Oxfordshire Research Ethics Committee (09/H0604/58).

### Consent and sample acquisition

We obtained PF from women aged 18 to 49 years old who were undergoing elective laparoscopy for suspected endometriosis due to pain symptoms and/or infertility investigation after informed consent. None of the women had been undergoing hormone treatment for at least 1 month at the time of surgery. The control patients had no history of endometriosis and were found to have no pelvic pathology (Table 1). Biological samples and clinical data were collected, processed, and stored according to World Endometriosis Research Foundation Endometriosis Phenome and Biobanking Harmonisation Project (WERF EPHect) protocols (30, 31, 32, 33). The exclusion criteria were malignancy, pregnancy, breastfeeding, and an inability to understand the study or the consent form.

**Table 1:** Endometriosis patient characteristics.

Sample	Age, years	ASRM staging	Menstrual cycle phase	Experimental group	Previous history of endometriosis	Other diagnosis
1	32	0	Proliferative	Control	No	None
2	39	0	Secretory	Control	No	None
3	34	0	Secretory	Control	No	None
4	38	0	Proliferative	Control	No	None
5	33	0	Proliferative	Control	No	None
6	24	0	Proliferative	Control	No	None
7	31	1	Proliferative	Stage I/II	No	None
8	28	1	Secretory	Stage I/II	Yes (1 previous surgery)	None
9	33	2	Proliferative	Stage I/II	Yes (1 previous surgery)	
10	46	1	Secretory	Stage I/II	No	None
11	29	2	Secretory	Stage I/II	No	
12	38	2	Secretory	Stage I/II	No	Benign polyp
13	32	1	Secretory	Stage I/II	Yes (1 previous surgery)	None
14	45	2	Secretory	Stage I/II	Yes (1 previous surgery)	Fibroid (2.5 cm)
15	36	2	Proliferative	Stage I/II	No	Adhesions, fimbrial cysts
16	36	1	Proliferative	Stage I/II	No	Simple cyst
17	34	1	Secretory	Stage I/II	No	
18	22	1	Proliferative	Stage I/II	No	No
19	26	1	Proliferative	Stage I/II	No	None
20	27	1	Secretory	Stage I/II	No	Adhesions
21	35	1	Proliferative	Stage I/II	Yes (1 previous surgery)	None
22	32	1	Proliferative	Stage I/II	Yes (1 previous surgery)	None
23	33	4	Proliferative	Stage III/IV	Yes (1 previous surgery)	None
24	40	3	Secretory	Stage III/IV	Yes (1 previous surgery)	None
25	43	4	Proliferative	Stage III/IV	Yes (1 previous surgery)	None
26	36	3	Proliferative	Stage III/IV	No	Simple endometrial cyst
27	48	3	Secretory	Stage III/IV	No	Cysts
28	38	3	Secretory	Stage III/IV	No	None

*Note:* ASRM = American Society for Reproductive Medicine.

A total of 28 women were included in the study (see Table 1). Endometriosis was staged according to the American Society for Reproductive Medicine (ASRM) classification (9). Cycle phases were self-reported and confirmed by histology of endometrial biopsy samples taken during the laparoscopy (34). In case of discordance, the histologic result was used.

For this exploratory study, only 1 mL of clear PF per patient was available due to the material demands of other research performed out under ENDOX. To reduce biological variation and in keeping with earlier studies on serum proteomics (35) and on exosomes isolated from small amounts of liquid (36), the samples of women with stage I and stage II endometriosis, and the samples of women with stage III and stage IV endometriosis were pooled after histologic confirmation of their menstrual cycle phase. This resulted in six experimental groups: control–proliferative, stage I/II–proliferative, stage III/IV–proliferative, control-secretory, stage I/II–secretory, and stage III/IV–secretory.

### Exosome isolation

**Figure 1:**
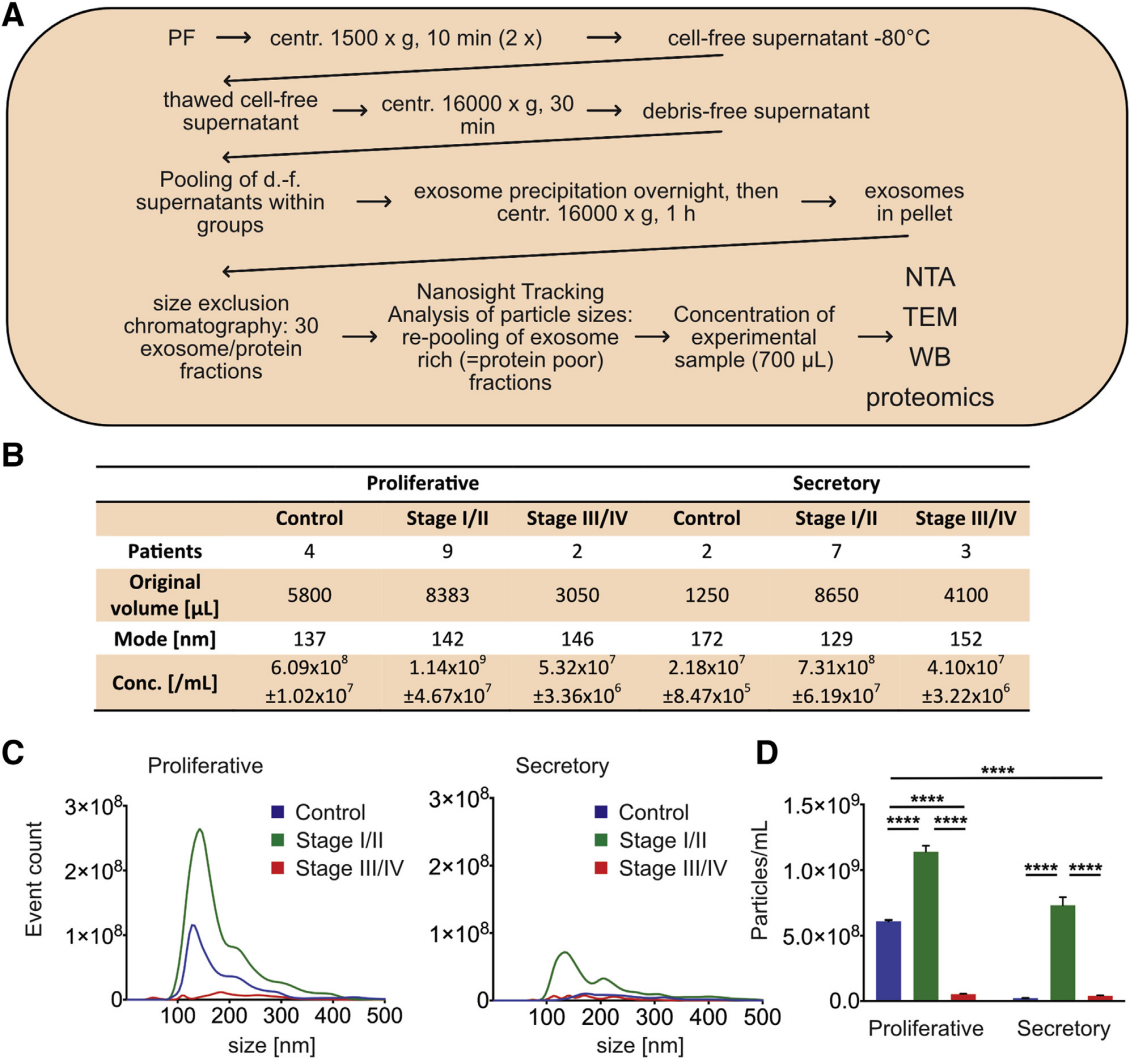
Isolation and characterization of exosomes from peritoneal fluid (PF). (**A**) Exosome isolation protocol. Peritoneal fluid (PF) was centrifuged twice to remove cells, and the supernatant was frozen for batch analysis. Upon thawing, the samples were spun to remove cell debris and larger, nonexosomal particles. The supernatants were pooled according to patient group to have sufficient material for downstream analysis. Exosomes were precipitated, and each pooled sample was fractionated by size exclusion chromatography. Fractions were analyzed for exosome and protein content, and the exosome-rich and protein-poor fractions were reunited as the experimental sample. The volume was adjusted to 700 μL. (**B**) Sample characteristics as per nanoparticle tracking analysis (NTA) analysis. The mode is the prevalent particle size, with exosome size ranging from 100–200 nm. (**C**) The size and concentration of exosomes within the groups in *B* measured by NTA. The analysis was done separately for proliferative and secretory cycle phases. The peaks indicate the presence of exosomes. (**D**) The comparison of exosome concentrations within samples shows statistically significant differences between cycle phases and disease stages. ****P<.0001, two-way analysis of variance with Tukey’s post-test.

Exosomes were isolated as described for placental perfusate before (37). Briefly, PF was put on ice at acquisition and then was centrifuged twice at 1,500 × *g* for 10 minutes at room temperature to remove cells (Fig. 1A). We and others have shown that this does not compromise exosome content or quality (37, 38). The pellet was discarded, and the cell-free PF supernatant was stored in 1-mL aliquots at −80°C until use.

Cell-free PF supernatants were thawed and centrifuged at 16,000 × *g* for 30 minutes to remove microvesicles and cell debris. Debris-free supernatants were pooled within the six experimental groups and were filtered through a 0.10-μm filter (Merck Millipore Ltd.). Exosomes were extracted using Exo-spin size-exclusion chromatography columns (Cell Guidance Systems) according to the manufacturer’s instructions. The samples were incubated at 4°C overnight with one half volume of Exo-spin buffer, then centrifuged for 1 hour at 16,000 × *g* and resuspended in 15 mL per group for column separation into 30 fractions at 500 μL (39). These fractions were analyzed for particle and protein content by nanoparticle tracking analysis, and exosome-rich/protein-poor fractions were reunited to obtain the experimental exosome sample (see the section on concentrating pf size-exclusion chromatography fractions).

### Nanoparticle tracking analysis

The particle content within the 30 fractions per group was measured using a NanoSight NS500 instrument (488 nm laser) with nanoparticle tracking analysis (NTA) software, version 3.1, Build 3.1.54 (Malvern Panalytical) and a high-sensitivity scientific complementary metal–oxide semiconductor (sCMOS) camera as previously described elsewhere (40). The samples were diluted with phosphate-buffered saline and infused into the sample chamber using a syringe pump module. The infusion rate was set so that events took 10 seconds to move across the screen. The camera brightness was kept at level 12. Five 90-second videos were recorded per sample and averaged to determine the particle concentration and sizes.

### Protein Measurement (Qubit)

The protein content of the 30 fractions per group was analyzed by a Qubit 4.0 Fluorometer using the Qubit Protein Assay (ThermoFisher Scientific).

### Concentrating PF size-exclusion chromatography fractions

**Supplemental Figure 1:**
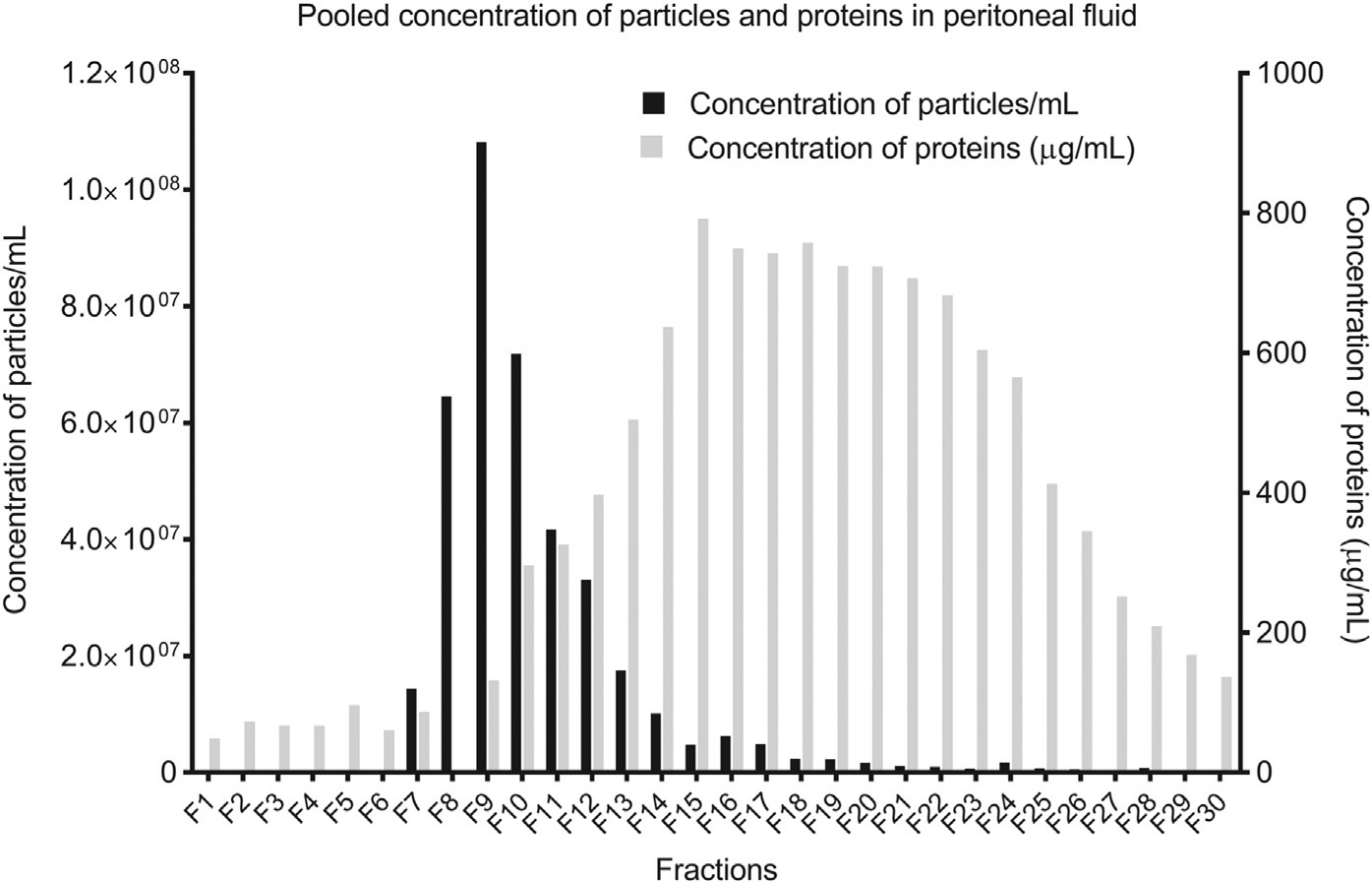
Protein versus particle content in fractionated sample. Plot showing the particle concentration per fraction as measured by nanoparticle tracking analysis (NTA) (black bars) compared with the free protein content (grey bars). The fractions with the highest number of particles (F8–F12) were selected, thus excluding the major portion of free (i.e. not exosome-bound) protein from downstream analysis.

The fractions with the highest exosome concentration and the least protein content were pooled and concentrated using an Amicon centrifugal filter unit (Ultra-4, 100 kDa; Millipore) and were reanalyzed by NTA (Supplemental Fig. 1, available online), taking the dilution factor and total sample volumes into consideration.

### Western Blot

The samples were tested for the presence of exosome protein markers using immunoblotting. Briefly, 10 μg of protein were loaded onto a NuPAGE Novex 4-12% Bis-Tris Gel (ThermoFisher Scientific/Invitrogen) then run in NuPAGE MOPS SDS Running Buffer (ThermoFisher Scientific/Invitrogen) at 150 V for 1 hour. The proteins were electroblotted for 1 hour at 25 V onto a methanol-activated polyvinylidene difluoride membrane (Bio-Rad Laboratories). The protein transfer was ascertained by Ponceau Red stain (Merck). Antibodies used for immunostaining were anti-CD9 (Abcam), anti-Alix (Cell Signaling Technology), and anti-syntenin (Abcam). The syntenin band was visualized using the EZ-ECL kit (Geneflow), and the Alix and CD9 bands were visualized using the Westar Supernova ECL kit (Cynagen) with the Gel-Box imaging system (Sygene), or on Kodak films according to the manufacturer’s instructions.

### Liquid Chromatography with Tandem Mass Spectrometry and Proteomic Analysis

A total of 9.7 μg of protein was used, and the volumes were equalized to 85 μL with deionized water. The samples were lysed by adding the same volume of radioimmunoprecipitation assay buffer (ThermoFisher Scientific/Pierce) and incubating on ice for 30 minutes. After lysis the samples were centrifuged for 10 minutes, and the clear supernatant was transferred into fresh tubes. The samples were reduced by the addition of 5 μL of 200 mM dithiothreitol (30 minutes at room temperature) and alkylated with 20 μL of 200 mM iodoacetamide (30 minutes at room temperature) followed by methanol-chloroform precipitation.

The pellets were resuspended in 6 M urea in 400 mM Tris-HCl, pH 7.8. Urea was diluted to 1 M using 400 mM Tris-HCl, pH 7.8, and the proteins were digested with trypsin in a ratio of 1:50 (overnight at 37°C). After acidification to a final concentration of 1% formic acid, the samples were desalted on Sola HRP SPE cartridges (ThermoFisher Scientific) and dried down in a SpeedVac.

The peptides were separated on an EASY spray column (ES803; ThermoFisher Scientific) and analyzed on a Dionex Ultimate 3000/Orbitrap Fusion Lumos platform (both ThermoFisher Scientific) using standard parameters as described elsewhere (41).

Mass spectrometry data were analyzed quantitatively with Progenesis QI for proteomics (v4.1) after a database search (Swiss-Prot human 03/2018) by Mascot (v2.5) using standard parameters: 10 ppm/0.5 Da mass tolerance (MS1/MS2), 1 missed cleavage site, fixed carbamidomethylation (C), variable deamidation (D, Q), and oxidation (M). The peptide false-discovery rate was adjusted to 1%, and the peptides with ion scores lower than 20 were discarded. A full list of proteins identified is provided in Supplemental Table 1 (available online).

### Transmission electron microscopy

For exosome visualization, a carbon-coated 300 mesh copper grid was glow discharged and then incubated on a 10-μL droplet of the sample for 2 minutes, blotted with filter paper, negatively stained with 2% uranyl acetate for 10 seconds, blotted, and air dried. Grids were imaged at an accelerating voltage of 120 kV in a FEI T12 transmission electron microscopy (TEM) using a Gatan OneView digital camera.

### Statistical analysis

To compare the concentrations of exosomes between groups, we performed two-way analysis of variance of disease stage and menstrual cycle phase with Tukey’s post-test for multiple comparisons (Prism 7, Graph Pad Software). *P*<.05 was considered statistically significant.

## Results

### Exosome concentrations in PF vary depending on disease stage and cycle phase

The NTA profiles of the pooled PF samples showed peaks at around 150 nm, consistent with the presence of exosomes in all sample groups (see Fig. 1B and C). Particles larger than 500 nm were absent, indicating an enriched exosome sample without contamination by microvesicles or apoptotic bodies. The stage I/II endometriosis group showed a higher concentration of exosomes in PF compared with the control group in both cycle phases (see Fig. 1D; *P*<.0001). Although the low sample number is reason for caution (see Fig. 1B), this likely reflects the more prominent involvement of inflammatory cells in stage I/II disease compared with stage III/IV endometriosis, which frequently shows deep endometriosis with lesions removed from direct contact with the PF.

The exosome concentration changed overall with the menstrual cycle phase within the groups (see Fig. 1D, *P*<.0001). In three groups it was so low that only the minimal number of valid NTA tracks (≥200) could be recorded (proliferative stage III/IV group; secretory control and stage III/IV groups); however, the particle mode size—the most prominent peak—was nevertheless similar among all groups (129–172 nm; see Fig. 1B), indicating that we were indeed observing exosomes.

**Figure 2:**
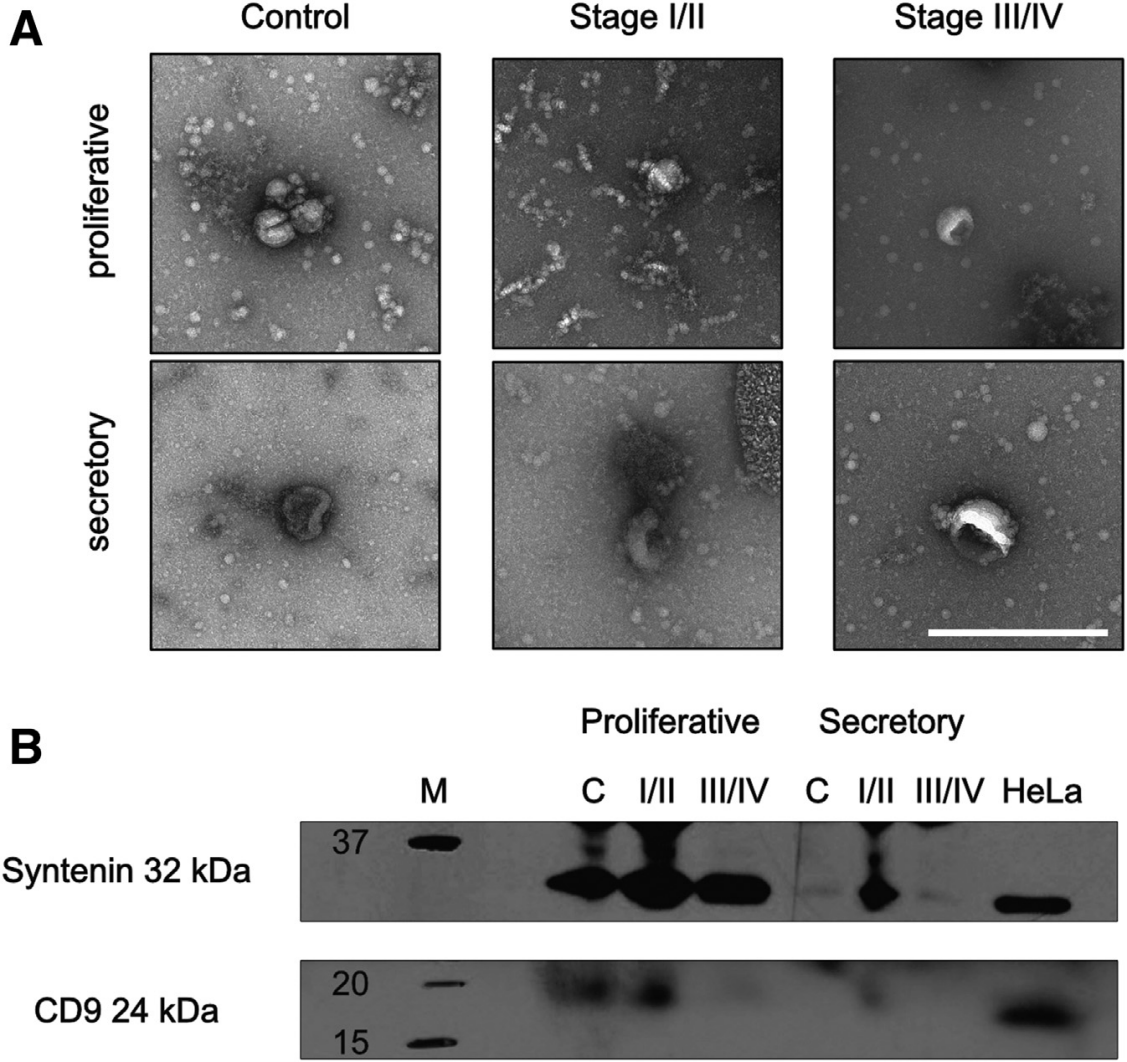
Identification of exosomes within the samples. (**A**) Transmission electron microscopy images showing the presence of exosomes in all six sample groups. Exosomes typically show an invaginated, cup-shaped morphology as a result of sample preparation. Scale bar: 500 nm. (**B**) Immunoblotting of the six experimental groups. Protein was extracted from the exosome preparations and blotted with antibodies against syntenin and CD9 as markers of exosomes. Bands demonstrate the presence of exosomes in all groups (C = control; I/II = stage I/II endometriosis; III/IV = stage III/IV endometriosis). HeLa whole cell lysate was used as positive control.

We confirmed the presence of exosomes by TEM and immunoblotting in all groups (Fig. 2). The electron microscopy images showed the typical invaginated cuplike appearance of exosomes at the expected size (see Fig. 2A). Immunoblotting of the samples indicated the presence of the exosome markers syntenin and CD9 in the preparations, although only a faint band was seen in the low-abundance samples mentioned earlier (see Fig. 2B). Alix was not detected in any of the samples (data not shown). This could point to the overall low protein amount or to an Alix-independent pathway of exosome biogenesis. In conclusion, exosomes are present within PF samples of women with and without endometriosis, independent of the cycle phase.

### Endometriosis-specific exosome proteins

**Figure 3:**
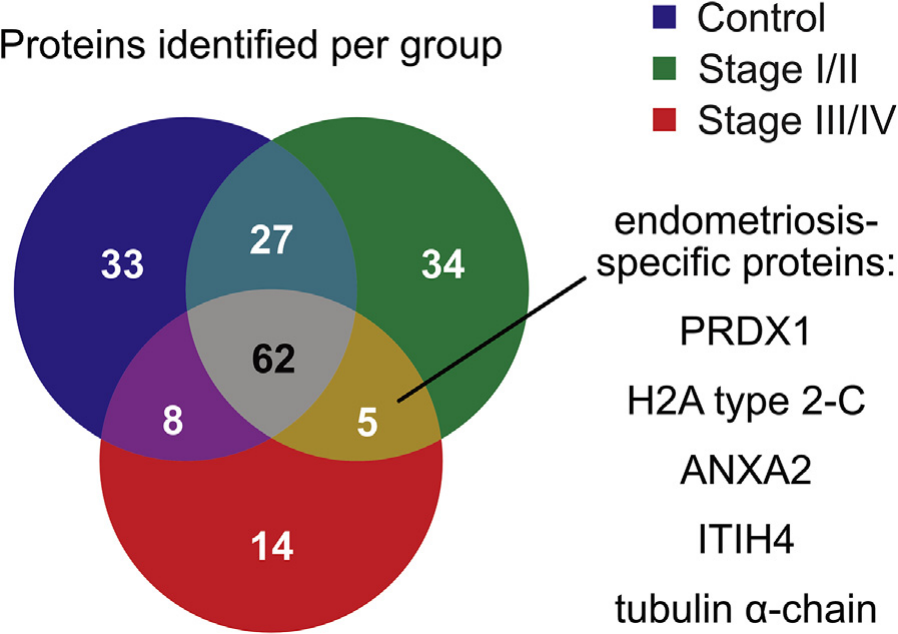
Proteomic analysis of peritoneal fluid (PF) exosome cargo revealing the distinct presence of endometriosis-associated exosomes. Exosomes isolated from the PF of patients and controls were analyzed by liquid chromatography with tandem mass spectrometry according to disease stage. This allowed for the detection of five proteins found in both disease groups but absent in controls. The diagram depicts the number of proteins found in all experimental groups.

We analyzed the pooled exosome samples by liquid chromatography with tandem mass spectrometry to investigate the exosome protein cargo of the different groups. Using label-free relative quantitation of proteins with at least two unique peptides identified and an false-discovery rate <1%, we detected proteins shared between all exosomes as well as distinct proteins present within each group (Fig. 3; see Supplemental Table 1). Overall, the exosome cargo analysis showed 62 proteins shared by the control and endometriosis groups regardless of menstrual phase, at a maximum reading depth of 150 proteins. A gene ontology analysis of cell compartment origin of these proteins and the comparison with the ExoCarta data set of exosome proteins (42) confirmed them as exosome-derived.

To define an endometriosis-specific exosome population, we combined the data of both cycle phases to average out cycle phase-specific markers in favor of an endometriosis-specific signature. Five proteins were found to be present within endometriosis samples (stage I/II and stage III/IV) that were absent from the control samples: (fragments of) peroxiredoxin-1 (PRDX1), histone H2A type 2-C, annexin A2 (ANXA2), inter-α-trypsin inhibitor heavy chain H4 (ITIH4) (fragment), and tubulin α-chain.

**Supplemental Figure 2:**
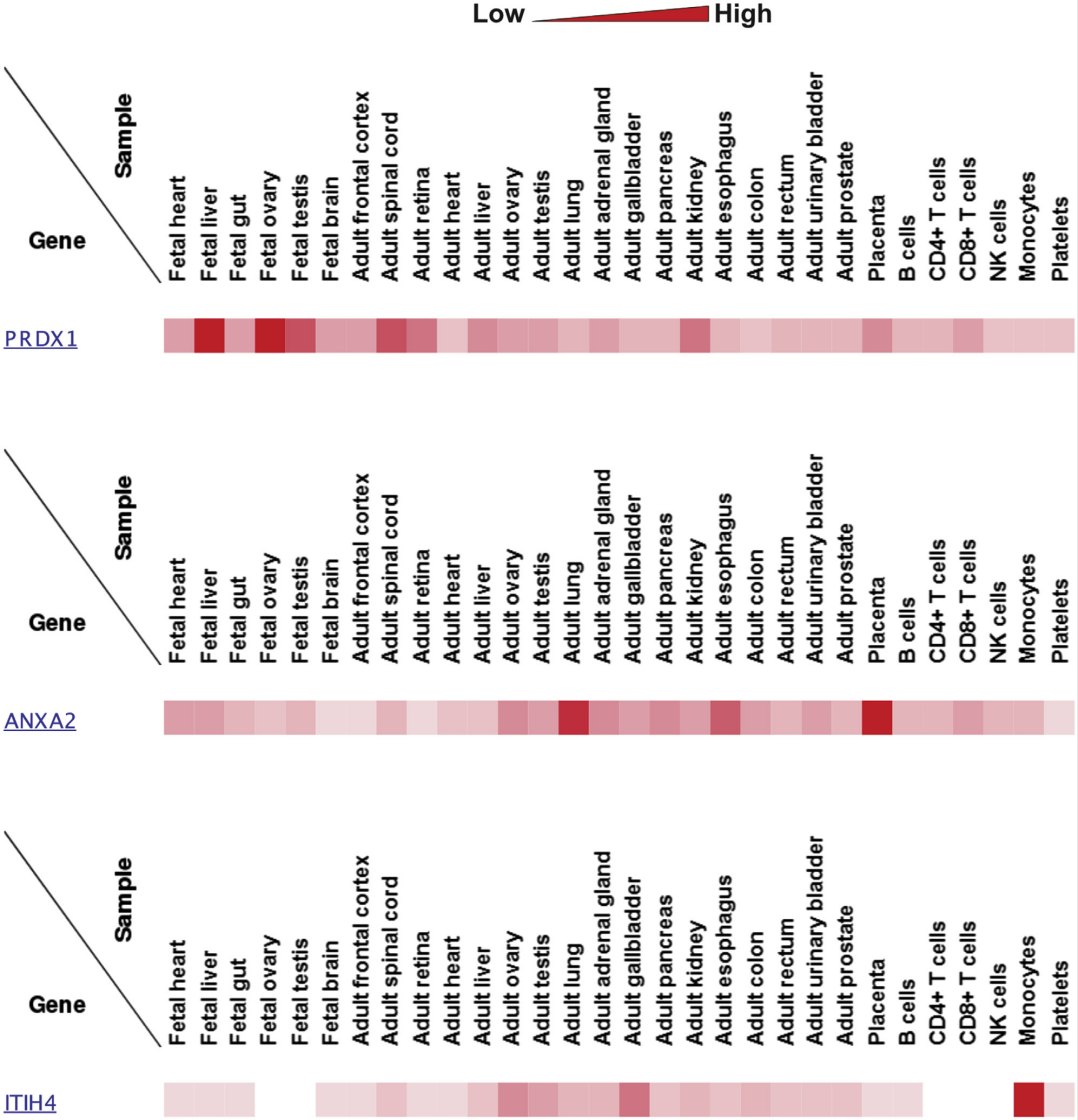
Human Proteome Map analysis of endometriosis-specific exosome protein expression. The exosome proteins found in endometriosis stage I/II and stage III/IV groups but not in controls were searched within the Human Proteome Map database (43) to indicate their origin.

We consulted the expression data from the Human Proteome Map (43) database to learn about the potential origin of the exosomes carrying these proteins. The database contains data for three out of the five proteins identified (Supplemental Fig. 2, available online), with PRDX1 expressed mainly in fetal tissues but also in placenta and kidney, ANXA2 in placenta and lung, and ITIH4 as predominantly expressed on monocytes. Thus, PRDX1 and ANXA2 are linked to placental/fetal development, while ITIH4 most likely derives from inflammatory monocytes prevalent in endometriosis.

Taken together, we directly identified exosomes in the PF of women. The exosome populations vary between nonendometriotic controls and endometriosis patients, and they carry proteins consistent with a potential role in the pathogenesis of endometriosis.

## Discussion

Endometriosis is characterized by ongoing, chronic inflammation in the peritoneum (44), and PF is rich in cytokines, prostaglandins, interleukin, and other growth factors. We show that exosomes are also part of this list of PF constituents.

It is thought that stage I/II disease especially contains “active” lesions involving increased angiogenesis and immune processes (45, 46). We found elevated numbers of exosomes in PF of women with ASRM stage I/II endometriosis, particularly during the proliferative phase of the menstrual cycle. As to the origin of these exosomes, it is conceivable that they were released from immune, mesothelial, or neuronal cells, from endometriotic lesions per se, or that they were retrogradely washed in during the last menstrual period. Exosomes from menstrual blood-derived stem cells (MenSCs) have been described previously as antiangiogenic (47) and anti-inflammatory (48), and promoting neurite outgrowth (49); however, our analysis of PF exosome protein cargo did not indicate a MenSCs origin.

Apart from proteins, exosome cargo comprises of microRNA, RNA (50), piwi-interacting RNA, transfer RNA fragments (16), DNA (51) and secondary messenger molecules (52) capable of affecting the gene expression and behavior of their target cells, such as phenocopying (53). Their investigation was beyond the scope of our study. However, we investigated the protein content of isolated exosomes. While 62 proteins were shared by exosomes from endometriosis patients and controls irrespective of menstrual phase, five proteins were found exclusively in endometriosis samples: (fragments of) PRDX1, histone H2A type 2-C, ANXA2, ITIH4 (fragment), and tubulin α-chain.

The PRDX1 gene is overexpressed in human malignancies, suggesting that PRDX1 may be a proto-oncogene, although in some studies PRDX1 has been shown to suppress tumors (54). ANXA2, an activator of matrix metallopeptidase (MMP-9) (55), is essential for degradation of the extracellular matrix of retrogradely menstruated endometrium by peritoneal macrophages (56). Similarly, the extracellular matrix of the peritoneal layer of mesothelial cells could be compromised, enabling ectopic endometrial lesions to attach and develop here. An earlier proteomics study showed ANXA2 dysregulation within the eutopic endometrium of endometriosis patients (57). Both PRDX1 and ANXA2 are elevated in stage I endometrial cancer (58), and ANXA2 has been suggested as a potential biomarker for recurrent endometrial cancer (59) and in ovarian cancer progression (regulation of β-catenin-driven epithelial-mesenchymal transition) (60). Thus, ANXA2 is already an established protein of interest in the field. Closer to endometriosis, ANXA2 has been implied in adenomyosis-associated dysmenorrhea (61). Furthermore, ANXA2 has been shown to correlate with gastric cancer growth and spreading, suggesting a role in metastasis (62); a similar role for ANXA2 could be envisaged in the establishment of endometriotic lesions.

An RNA expression of ITIH4 has been detected only in the liver (63), and it seems to be up-regulated during surgical trauma, which would explain its presence within our sample cohort. We alternatively assume an inflammatory component as the origin of specific exosomes in endometriosis, such as inflammatory monocytes. Moreover, ITIH4 has been suggested as a serum biomarker for recurrent pregnancy loss patients (64). On the other hand, histone and tubulin are constituents of normal cell machinery and are less likely endometriosis-associated proteins, although the possibility that the presence of these proteins reflects an altered cell machinery cannot be excluded.

We have shown increased exosome concentration in stage I/II disease in keeping with the ongoing inflammatory response during endometriosis, and we saw a decrease in stage III/IV disease. Genetic studies have recently indicated that stages I/II and III/IV endometriosis are distinct in their pathogenesis (65). Here, the PF-exosome concentration may be a useful biomarker to separate these two entities.

It can be speculated that exosomes are priming the peritoneal mesothelial cell layer for implantation of endometriotic tissue, similar to tumor exosomes creating the premetastatic niche (66). Exosomes may also be involved in endometriosis-associated pain either by promoting neurite outgrowth (67) or transporting neurotransmitters (49).

### Clinical implications

Exosomes are of increasing interest in the pathophysiology of endometriosis, and a recent review by Schjenken et al. (68) asked that “future studies should examine the composition of these exosomes,” referring to exosomes produced by endometriotic lesions and released into the PF. We believe to have made the first step in that direction. The successful isolation of exosomes directly from PF and the analysis presented here suggest proteins specifically found in exosomes from endometriosis patients as biomarkers of the disease. However, similar to other conditions (68), the systemic presence of endometriosis-specific exosomes will have to be confirmed to use, for instance, a simple blood test to confirm the presence of the disease.

### Research implications

Exosomes have the potential to direct tumor metastasis in cancer, and their role in endometriosis is as yet unclear. However, their diverse nature could provide an explanation as to why endometriosis occurs in some women and not in others, given that retrograde menstruation (the most commonly cited source of endometriotic cells) is very common. We have shown the presence of exosomes in PF, but more experimental research is needed to functionally characterize the role of exosomes in endometriosis.

### Strengths and limitations

Although challenging in its approach, this is the first analysis of exosomes directly isolated from PF as opposed to exosomes derived from cultured tissue. A caveat is that only a small volume (≤1 mL) of PF per patient was available for analysis, which required the pooling of samples within groups to reach the amounts necessary for analysis. Thus, we cannot ascribe our findings to individual patients. We were able to use PF samples only of patients who had not taken hormones for at least 1 month before surgery and whose PF samples were of a large enough volume to allow for their use in our experiments once the amounts required for other research under ENDOX had been stored according to the study protocol. This left us with samples from 28 patients only, whom we further divided according to cycle phase and disease stage to avoid confounding our results. This limitation introduces a potential bias in that a few additional patients in the control group could have resulted in a different picture.

## Conclusion

Exosomes in the PF of women present a worthwhile target of investigation. Future larger scale studies will enable us to focus on particular aspects of exosome involvement in the disease, which should give further insight into the pathogenesis of endometriosis. Multicenter collaborative efforts such as supported by WERF and performed according to EPHect standards will be instrumental in this effort.
