# Supplementary material for: Characterization of exosomes in peritoneal fluid of endometriosis patients
Source: Fertil Steril. 2020 Feb;113(2):364–373.e2. doi: 10.1016/j.fertnstert.2019.09.032 (PMC7057257; doi:10.1016/j.fertnstert.2019.09.032)
Supplement: Supplemental Table 1 [file mmc1.docx]

**Supplemental Table 1: Exosomal proteins identified by mass spectrometry (LS-MS/MS) within the different groups.**

| Proliferative | | | Secretory | | |
| --- | --- | --- | --- | --- | --- |
| **Control** | **Stage I/II** | **Stage III/IV** | **Control** | **Stage I/II** | **Stage III/IV** |
| Glial fibrillary acidic protein (Fragment) | Glial fibrillary acidic protein (Fragment) | Glial fibrillary acidic protein (Fragment) | Glial fibrillary acidic protein (Fragment) | Glial fibrillary acidic protein (Fragment) | Hornerin |
| Hornerin | Isoform 2 of Neurofilament heavy polypeptide | Hornerin | Hornerin | Hornerin | Junction plakoglobin |
| Complement C3 | Hornerin | Desmoplakin | Desmoplakin | Desmoplakin | Desmoplakin |
| Desmoplakin | Desmoplakin | Filaggrin-2 | Filaggrin-2 | Junction plakoglobin | Desmoglein-1 |
| Junction plakoglobin | Desmoglein-1 | Complement C3 | Junction plakoglobin | Desmoglein-1 | Complement C3 |
| Filaggrin-2 | Filaggrin-2 | Junction plakoglobin | Desmoglein-1 | Filaggrin-2 | Filaggrin-2 |
| Apolipoprotein B-100 | Complement C3 | Desmoglein-1 | Complement C3 | Protein-glutamine gamma-glutamyltransferase E | Glyceraldehyde-3-phosphate dehydrogenase |
| Desmoglein-1 | Junction plakoglobin | Complement C4-A | Isoform 2 of Serpin B12 | Glyceraldehyde-3-phosphate dehydrogenase | Alpha-1-antichymotrypsin |
| Complement C4-B | Protein-glutamine gamma-glutamyltransferase E | Glyceraldehyde-3-phosphate dehydrogenase | Protein-glutamine gamma-glutamyltransferase E | Isoform 1B of Desmocollin-1 | Immunoglobulin heavy constant mu |
| Alpha-2-macroglobulin | Isoform 2 of Serpin B12 | Alpha-2-macroglobulin | Glyceraldehyde-3-phosphate dehydrogenase | Complement C3 | Apolipoprotein A-I |
| Isoform 2 of Inter-alpha-trypsin inhibitor heavy chain H4 | Isoform 2 of Arginase-1 | Desmocollin-1 | Serpin B3 | Alpha-2-macroglobulin | Alpha-2-macroglobulin |
| Alpha-1-antichymotrypsin | Serpin B3 | Angiotensinogen | Serpin B4 | Actin, cytoplasmic 1 | Histone H2A type 1 |
| Actin, cytoplasmic 1 | Serpin B4 | Isoform 2 of Serpin B12 | Actin, cytoplasmic 1 | Serpin B12 | Histone H2A type 2-C |
| Angiotensinogen | Glyceraldehyde-3-phosphate dehydrogenase | Actin, cytoplasmic 2 | Actin, aortic smooth muscle | Annexin A1 | Immunoglobulin lambda-like polypeptide 5 |
| Protein-glutamine gamma-glutamyltransferase E | Actin, cytoplasmic 1 | Suprabasin | Isoform 1B of Desmocollin-1 | Caspase-14 | Trypsin-1 (Fragment) |
| cDNA FLJ55673, highly similar to Complement factor B (EC 3.4.21.47) | Isoform 2 of Annexin A2 | Inter-alpha-trypsin inhibitor heavy chain H2 | Isoform 2 of Annexin A2 | Isoform 1 of Plakophilin-1 | Isoform 1B of Desmocollin-1 |
| Inter-alpha-trypsin inhibitor heavy chain H1 | Bleomycin hydrolase | Antithrombin-III | Filaggrin | Serpin B3 | Complement C4-B |
| Immunoglobulin kappa constant | Isoform 1B of Desmocollin-1 | Alpha-1-antichymotrypsin | Isoform 1 of Protein POF1B | Serpin B4 | Caspase-14 |
| Inter-alpha-trypsin inhibitor heavy chain H2 | Filaggrin | Protein-glutamine gamma-glutamyltransferase E | Annexin A1 | Isoform 2 of Arginase-1 | Immunoglobulin kappa constant |
| Isoform 1B of Desmocollin-1 | Alpha-2-macroglobulin | Histone H2A type 2-A | Epiplakin | Catalase | Inter-alpha-trypsin inhibitor heavy chain H2 |
| Hemopexin | Immunoglobulin kappa constant | Histone H2A type 1-B/E | Isoform 2 of Plectin | Filaggrin | Hemopexin |
| Serpin B12 | Alpha-1-antichymotrypsin | Histone H2A.Z | Immunoglobulin kappa constant | Heat shock protein beta-1 | Actin, cytoplasmic 1 |
| Apolipoprotein A-I | Complement C4-B | Plasma protease C1 inhibitor | Isoform 2 of Arginase-1 | Immunoglobulin heavy constant mu (Fragment) | Fibrinogen gamma chain |
| Glyceraldehyde-3-phosphate dehydrogenase | Suprabasin | Isoform 2 of Inter-alpha-trypsin inhibitor heavy chain H4 | Complement C4-B | Annexin A2 | Annexin A2 |
| Alpha-1-antitrypsin | Isoform 1 of Plakophilin-1 | Isoform 2 of Arginase-1 | Alpha-enolase | Immunoglobulin kappa constant | Alpha-1-antitrypsin |
| Ceruloplasmin | Plasma protease C1 inhibitor | Heat shock protein beta-1 | Caspase-14 | Proteasome subunit alpha type | Lysozyme |
| Antithrombin-III | Catalase | Immunoglobulin heavy constant alpha 1 (Fragment) | Pyruvate kinase PKM | Complement C4-B | Prolactin-inducible protein |
| Isoform 2 of Arginase-1 | Inter-alpha-trypsin inhibitor heavy chain H2 | Dermcidin | Histidine ammonia-lyase | Alpha-enolase | Immunoglobulin heavy constant alpha 1 (Fragment) |
| Catalase | Caspase-14 | Hemopexin | Heat shock cognate 71 kDa protein | Dermcidin | Hemoglobin subunit gamma-1 |
| Isoform 2 of Annexin A2 | Protein-glutamine gamma-glutamyltransferase K | Catalase | Heat shock-related 70 kDa protein 2 | Isoform 2 of Dermcidin | Hemoglobin subunit delta |
| Lipocalin-1 | Angiotensinogen | Zinc-alpha-2-glycoprotein | 78 kDa glucose-regulated protein | Peroxiredoxin-2 | Antithrombin-III |
| Gelsolin | Zinc-alpha-2-glycoprotein | Isoform 2 of Annexin A2 | Isoform 2 of Heat shock 70 kDa protein 1A | Peroxiredoxin-1 | Desmocollin-3 |
| Prolactin-inducible protein | Pyruvate kinase PKM | Apolipoprotein B-100 | Apolipoprotein B-100 | Suprabasin | Apolipoprotein B-100 |
| Trypsin-1 | Immunoglobulin heavy constant mu | Ceruloplasmin | Gasdermin-A | Galectin-7 | Filaggrin |
| Caspase-14 | Inter-alpha-trypsin inhibitor heavy chain H4 (Fragment) | Bleomycin hydrolase | Alpha-2-macroglobulin | Antithrombin-III | Ceruloplasmin |
| Plasma protease C1 inhibitor | Antithrombin-III | Vitamin D-binding protein | Histone H2A type 2-A | Gamma-glutamylcyclotransferase | Plasma protease C1 inhibitor |
| Vitamin D-binding protein | Isoform 2 of Triosephosphate isomerase | Isoform 1 of Protein POF1B | Histone H2A type 1 | Bleomycin hydrolase | Isoform 3 of Pyruvate kinase PKM |
| Isoform 1 of Plakophilin-1 | Ceruloplasmin | Peroxiredoxin-2 | Isoform 1 of Plakophilin-1 | Zinc-alpha-2-glycoprotein | Isoform 2 of Arginase-1 |
| Complement C5 | Annexin A1 | Peroxiredoxin-1 | Suprabasin | Heat shock cognate 71 kDa protein | Fibrinogen beta chain |
| Alpha-2-antiplasmin | Calmodulin-like protein 5 | Galectin-7 | Protein-glutamine gamma-glutamyltransferase K | 78 kDa glucose-regulated protein | Peroxiredoxin-1 (Fragment) |
| Protein-glutamine gamma-glutamyltransferase K | Serpin A12 | Protein S100-A8 | Isoform 3B of Desmocollin-3 | Calmodulin-like protein 5 | Peroxiredoxin-2 |
| Serpin B3 | Heat shock protein beta-1 | Trypsin-1 | Heat shock protein beta-1 | Pyruvate kinase PKM | Complement component C9 |
| Serotransferrin | Isoform 2 of Dermcidin | Inter-alpha-trypsin inhibitor heavy chain H1 | Galectin-7 | Isoform 2 of Triosephosphate isomerase | Protein-glutamine gamma-glutamyltransferase E |
| Isoform 2 of Heat shock 70 kDa protein 1A | Dermcidin | Alpha-1-antitrypsin | Putative elongation factor 1-alpha-like 3 | Gasdermin-A | Dermcidin |
| Lysozyme | Putative elongation factor 1-alpha-like 3 | Serotransferrin | Catalase | Histone H2A type 1 | Inter-alpha-trypsin inhibitor heavy chain H4 (Fragment) |
| Galectin-7 | Isoform 3B of Desmocollin-3 | Apolipoprotein A-I | Fatty acid-binding protein, epidermal | Histone H2A type 2-C | Isoform 2 of Serpin B3 |
| Dermcidin | Peroxiredoxin-1 (Fragment) | Immunoglobulin kappa constant | Apolipoprotein A-I | Histidine ammonia-lyase | Transthyretin |
| Immunoglobulin heavy constant mu | Gasdermin-A | Caspase-14 | Alpha-1-antitrypsin | Elongation factor 2 |  |
| Histone H4 | Lactotransferrin (Fragment) | Serpin A12 | Gelsolin | Isoform 3B of Desmocollin-3 |  |
| Fibrinogen beta chain | Protein S100-A9 | Gamma-glutamylcyclotransferase | Antithrombin-III | Immunoglobulin heavy constant alpha 2 (Fragment) |  |
| Isoform 1 of Protein POF1B | Immunoglobulin lambda constant 2 | Tubulin alpha chain | Alpha-1-antichymotrypsin | Cystatin-A |  |
| Annexin A1 | Histidine ammonia-lyase | Serpin B3 | Protein S100-A8 | Isoform 2 of Dermokine |  |
| Complement component C9 | Apolipoprotein B-100 | Isoform 3B of Desmocollin-3 | Fructose-bisphosphate aldolase | Angiotensinogen |  |
| Immunoglobulin heavy constant alpha 1 (Fragment) | Heat shock cognate 71 kDa protein | Prolactin-inducible protein | Isoform C of Prelamin-A/C | Alpha-1-antichymotrypsin |  |
| Fibrinogen gamma chain | 78 kDa glucose-regulated protein | Protein-glutamine gamma-glutamyltransferase K | Glutathione S-transferase P | Isoform ADelta10 of Prelamin-A/C |  |
| Heat shock protein beta-1 | Alpha-enolase | ATP synthase subunit beta, mitochondrial | Peroxiredoxin-2 | Lactotransferrin (Fragment) |  |
| Desmocollin-3 | Isoform 1 of Protein POF1B | Isoform 2 of Triosephosphate isomerase | Peroxiredoxin-1 | Elongation factor 1-alpha 2 |  |
| Putative elongation factor 1-alpha-like 3 | Proteasome subunit alpha type | Histone H4 | Immunoglobulin lambda-like polypeptide 5 | Protein S100-A8 |  |
| Gasdermin-A | Protein S100-A8 | Fatty acid-binding protein, epidermal | Zinc-alpha-2-glycoprotein | Immunoglobulin lambda-like polypeptide 5 |  |
| Apolipoprotein C-I | Cystatin-A | Transthyretin | Prolactin-inducible protein | Zymogen granule protein 16 homolog B |  |
| Protein AMBP | Fatty acid-binding protein, epidermal | Complement component C9 | ITIH4 protein | Cathepsin D |  |
| Tubulin alpha-1B chain | Complement component C9 | Plakophilin-1 | Bleomycin hydrolase | Tubulin alpha chain |  |
| Zinc-alpha-2-glycoprotein | Immunoglobulin heavy constant alpha 2 (Fragment) | Cathepsin D | Dermcidin | Gamma-glutamyl hydrolase |  |
| Bleomycin hydrolase | Hemopexin | Polymeric immunoglobulin receptor | Cathepsin D | Tubulin beta chain |  |
| 60 kDa heat shock protein, mitochondrial | Thioredoxin | Alpha-2-antiplasmin | Elongation factor 2 | Epiplakin |  |
| Peroxiredoxin-2 | Lysozyme C | Heat shock cognate 71 kDa protein | Immunoglobulin heavy constant alpha 2 (Fragment) | Isoform 2 of Plectin |  |
| Extracellular glycoprotein lacritin | Tubulin alpha chain | 78 kDa glucose-regulated protein | Isoform 4 of Extracellular matrix protein 1 | Isoform 2 of Histone H2B type 2-F |  |
| Suprabasin | Peroxiredoxin-2 | Alpha-enolase | Triosephosphate isomerase | Histone H4 |  |
| Alpha-enolase | Transthyretin |  | Gamma-glutamylcyclotransferase | HCG1745306, isoform CRA_a |  |
| Cathepsin D | Apolipoprotein A-I |  | Immunoglobulin heavy constant gamma 1 | Lysozyme C |  |
| Isoform 2 of Fibrinogen alpha chain | Alpha-1-antitrypsin |  | Immunoglobulin heavy constant gamma 2 | Prolactin-inducible protein |  |
| Isoform M1 of Pyruvate kinase PKM | Proteasome subunit beta type-5 |  | Zymogen granule protein 16 homolog B | Fibrinogen gamma chain |  |
| Immunoglobulin heavy constant gamma 3 (Fragment) | Prolactin-inducible protein |  | Carboxypeptidase A4 | Fatty acid-binding protein, epidermal |  |
| Transthyretin | Cathepsin D |  | Fibrinogen gamma chain | Thioredoxin |  |
| Isoform 2 of Triosephosphate isomerase | Cystatin-M |  | Tubulin alpha-1B chain | Proteasome subunit alpha type-7 |  |
| Complement component C6 | Gelsolin |  | Polyubiquitin-B | Apolipoprotein A-I |  |
| Alpha-2-HS-glycoprotein | Proteasome subunit alpha type-7 |  | Aminopeptidase | Fructose-bisphosphate aldolase |  |
|  | F-box only protein 50 |  | Histone H3.1 | Calpain-1 catalytic subunit |  |
|  | Zymogen granule protein 16 homolog B |  | Proteasome subunit beta type-5 | Serotransferrin |  |
|  | Isoform 4 of Extracellular matrix protein 1 |  | F-box only protein 50 | Alpha-1-antitrypsin |  |
|  | Glutathione S-transferase P |  | Protein S100-A14 | Cornulin |  |
|  | Isoform ADelta10 of Prelamin-A/C |  | Isoform Long of Proteasome subunit alpha type-1 | F-box only protein 50 |  |
|  | Alpha-2-antiplasmin |  | Transthyretin | Apolipoprotein B-100 |  |
|  | Gamma-glutamylcyclotransferase |  | Glutaredoxin-1 | Haptoglobin |  |
|  | Immunoglobulin heavy constant gamma 1 (Fragment) |  | Trypsin-1 | Histone H1.2 |  |
|  | Fibrinogen gamma chain |  | Isoform 5 of Tropomyosin alpha-3 chain | Histone H1.4 |  |
|  | Immunoglobulin kappa variable 3-20 |  | Myosin-9 | Isoform 1 of Vinculin |  |
|  | RPS27A protein |  | Tubulin beta chain | Calmodulin-like protein 3 |  |
|  | Protein S100-A16 |  | Cystatin-A | Immunoglobulin heavy constant gamma 1 (Fragment) |  |
|  | Ezrin |  | Purine nucleoside phosphorylase | Isoform 4 of Gelsolin |  |
|  | Ribonuclease 7 |  | Serpin A12 | Protein S100-A9 |  |
|  | Calmodulin-like protein 3 |  | Ceruloplasmin | Alpha-2-HS-glycoprotein |  |
|  | Myosin-9 |  | Histone H1.4 | Protein-glutamine gamma-glutamyltransferase K |  |
|  |  |  | Histone H4 | Isoform 2 of Extracellular matrix protein 1 |  |
|  |  |  | Nucleoside diphosphate kinase B | Hemopexin |  |
|  |  |  | Lipocalin-1 | Puromycin-sensitive aminopeptidase |  |
|  |  |  | Proteasome subunit alpha type | Trypsin-1 |  |
|  |  |  | Phospholipase B-like 1 | Peptidyl-prolyl cis-trans isomerase A |  |
|  |  |  | Inter-alpha-trypsin inhibitor heavy chain H2 | Protein S100-A16 |  |
|  |  |  | Insulin-degrading enzyme | Cystatin-B |  |
|  |  |  | Protein S100-A16 | Isoform 2 of Alpha-2-antiplasmin |  |
|  |  |  | Inter-alpha-trypsin inhibitor heavy chain H1 | Isoform 2 of Alpha-2-macroglobulin-like protein 1 |  |
|  |  |  | Protein S100-A9 | Isoform 1 of Protein POF1B |  |
|  |  |  | Hemoglobin subunit alpha | Inter-alpha-trypsin inhibitor heavy chain H2 |  |
